# Educational Attainment, Decision-Making Preferences, and Interest in Evidence-Based Diabetes Prevention among Women with a History of Gestational Diabetes Mellitus

**DOI:** 10.1089/whr.2020.0116

**Published:** 2021-04-27

**Authors:** Hemali Panchal, Norman Turk, Tannaz Moin, Carol M. Mangione, Amanda Vu, Sarah Amaya, Keith C. Norris, Obidiugwu Kenrik Duru

**Affiliations:** ^1^ Department of Internal Medicine, University of California, Davis, Sacramento, California, USA.; ^2^ Division of General Internal Medicine and Health Services Research, University of California, Los Angeles, Los Angeles, California, USA.; ^3^ Division of Endocrinology, Diabetes and Metabolism, University of California, Los Angeles, Los Angeles, California, USA.; ^4^ HSR&D Center for the Study of Healthcare Innovation, Implementation and Policy, VA Greater Los Angeles Health System, Los Angeles, California, USA.

**Keywords:** education, gestational diabetes, lifestyle intervention, medical decision-making, metformin

## Abstract

***Background:*** The Diabetes Prevention Program (DPP) showed that lifestyle change or metformin is equally efficacious in preventing diabetes in women who have had gestational diabetes mellitus (GDM). Few studies have investigated the relationship between education and willingness to engage in either intervention and between education and preferred decision-making style.

***Methods:*** Within a large health system, we surveyed insured women 18–64 years old with a history of GDM, identified through the electronic health record. We estimated preference for decision-making style and interest in DPP lifestyle change and/or metformin by educational level, using multivariate logistic regression models controlling for age, race, and ethnicity.

***Results:*** Our sample (*n* = 264) was 36% Latino, 29% Asian, 28% non-Latino white, and 5% African American, with a mean age of 37 years. In terms of education, 31% had a postgraduate degree, 41% were college graduates, and 29% did not graduate from college. In multivariate analyses, willingness to engage in either intervention did not vary by education. Women who did not graduate from college were more likely to leave medical decisions to their provider (*p* = 0.004) compared to women with a college or postgraduate degree. However, regardless of education, over 80% of women preferred to make medical decisions themselves or jointly with their provider.

***Conclusions:*** Most women prefer to play an active role in their own medical decisions and have an interest in both evidence-based diabetes prevention strategies. This suggests that shared decision-making is appropriate for many women with a history of GDM and different levels of educational attainment.

## Introduction

Gestational diabetes mellitus (GDM) affects ∼2%–10% of all pregnancies in the United States and is more common among women with lower educational attainment, women of color, and with advancing age.^1–3^ About 20% of women who develop GDM subsequently progress to type 2 diabetes.^4^ However, rates of postpartum screening for persistent hyperglycemia are low among women with a history of GDM. Only about 10% of women with GDM receive follow-up screening between 6 and 12 weeks after delivery, and only 25% are screened in studies looking out to 12 months after delivery.^5,6^ The Diabetes Prevention Program (DPP) randomized trial demonstrated that among overweight/obese women with a history of GDM and prediabetes, lifestyle change (dietary modifications/exercise) resulting in loss of 5%–7% body weight or metformin is similarly effective in preventing or delaying type 2 diabetes.^7^ However, real-world uptake of evidence-based options for diabetes prevention, including either intensive lifestyle changes (*i.e.*, DPP) or metformin, is less than optimal. Few studies have described the factors associated with a preferred strategy for type 2 diabetes prevention among women with a GDM history, especially when women are offered the choice of DPP lifestyle change, metformin, both, or neither.

For overweight/obese women with a history of GDM, the decision to pursue lifestyle intervention versus metformin for type 2 diabetes prevention may be largely driven by individual preferences. Women often have competing demands, including childcare, which may impact their ability to prioritize management of their own health and engagement in preventive care services. Their social support systems, risk perception, and ongoing psychosocial stressors are among many important factors influencing their real-world decision-making.^8–12^ Shared decision-making (SDM), defined as a deliberative dialog weighing reasonable treatment options, is a potentially attractive option for this patient population.^13^ While there is a paucity of literature on SDM in women with GDM, our team has shown that SDM with a health professional leads to greater uptake of both DPP lifestyle change and metformin, as well as weight loss at 12 months follow-up, among overweight/obese patients with prediabetes compared to control patients receiving usual care.^14^ To our knowledge, there is one other published study examining SDM in this population focused on the timing of delivery during a GDM pregnancy,^15^ but none to date describing the willingness to engage in SDM during the postpartum period.^16–18^ In addition, little is known about the decision-making preferences of women with a GDM history who have different levels of educational attainment.

The goal of this study was to investigate within an insured patient population whether the educational attainment of women with a GDM history is associated with their preferred decision-making approach in the health care setting, to assess whether a future SDM diabetes prevention intervention might be acceptable in this patient population. Specifically, we measured responses to the Control Preference Scale, which is considered an antecedent to the decision-making process, because patients who indicate an interest in contributing to their medical decisions are more willing to engage in SDM.^19,20^ In addition, we sought to assess if there was an association between educational attainment and which interventions, if any, women would be interested in: pharmacological (*i.e.*, metformin) and/or lifestyle change (*i.e.*, DPP curriculum). We hypothesized that compared to women with less than a college degree, women who had graduated from college or had some postcollege education would be more interested in contributing to the decision about type 2 diabetes prevention options and would also be more interested in both of the evidence-based prevention interventions.

## Methods

### Setting and participants

Using the University of California, Los Angeles (UCLA) electronic health record (EHR), we identified women between 18 and 50 years of age with a history of GDM and body mass index (BMI) ≥24 kg/m^2^ (BMI ≥22 kg/m^2^ if Asian American) as potential survey participants. We identified GDM diagnoses using billing codes, specifically International Classification of Diseases (ICD)-9 (648.83 or V12.21) or ICD-10 (O24.x); these diagnoses could have been made any time before the survey. We specifically limited the sample to women with these BMI values to align with the lower limit for participation in a Centers for Disease Control and Prevention (CDC)-recognized DPP, at the time of the study. We excluded women with known diabetes (*i.e.*, diagnosis of type 1 or type 2 diabetes recorded in the EHR, any Hemoglobin A1c >6.4%, or any current antihyperglycemic medication use). We used a multistep recruitment process, first reaching out through the EHR to the primary care physicians of potential participants to confirm that they met our inclusion criteria. We then mailed recruitment letters to eligible women inviting them to participate in the survey, which included a prepaid postcard they could return if they preferred not to be further contacted. Between May 2019 and December 2019, bilingual research assistants fluent in English and Spanish called eligible participants to obtain informed consent, confirm that they had never been diagnosed with type 2 diabetes, and administer the survey. Participants were compensated for their time with a $40 gift card for completing the survey. The study was approved by the UCLA Institutional Review Board (No. 18-001058).

### Questionnaire items/variables

The preamble to the study questionnaire introduced the DPP as a group behavioral change program endorsed by the CDC and led by a trained lifestyle coach, with a curriculum of 24 classes conducted over 1 year. The preamble also noted that metformin is an oral medication typically used in the treatment of diabetes but that it can also prevent diabetes, and also that research has demonstrated it works as well as lifestyle change in preventing diabetes for women with a history of GDM.

The study questionnaire had a single item to assess the level of agreement participants had with their prior GDM diagnosis (“How much do you disagree or agree with the statement ‘I don't think I truly had gestational diabetes?”). We included items assessing interest in attending the 12-month DPP program and/or taking metformin for diabetes prevention, with four possible initial responses (“Not interested,” “Somewhat interested,” “Moderately interested,” or “Very interested”), although for analytic purposes we combined the latter three options as having any interest in diabetes prevention.

We also included the Control Preference Scale for decision-making^19^ using a 5-point Likert scale: “I prefer to make the final decision,” “I prefer to make the final decision after seriously considering my doctor's opinion,” “I prefer that my doctor and I share responsibility for deciding which treatment is best,” “I prefer my doctor make the final treatment decision, but only after my doctor has seriously considered my opinion,” or “I prefer to leave all treatment decisions to my doctor.” For analytic purposes, we combined the first two options as the participant choosing to primarily make the decision themselves, combined the latter two options as the participant preferring that the provider make the decision, and kept the middle option of equally shared responsibility for the decision, resulting in three outcome categories.

Women were asked to self-report the presence of a family history of diabetes, the number of children they had, their educational attainment (less than college graduate, graduated college, or postgraduate degree), race, and ethnicity. Due to small sample size, we were unable to include women with less than a high school degree as a comparison group. While we did not have specific details on DPP insurance coverage for each survey respondent, we asked a theoretical question about how much each would be willing to pay out of pocket for the DPP. We collected information on age and BMI from the UCLA Health EHR.

### Statistical analyses

We constructed separate multivariate ordinal logistic regressions to examine the relationship between educational attainment and decision-making preferences, interest in taking metformin, and interest in participating in DPP classes, all adjusted for age, race, and ethnicity. We examined bivariate associations between educational attainment and agreement with the GDM diagnosis, as well as bivariate associations between educational attainment and the amount women would be willing to pay out of pocket for the DPP. All analyses were done using SAS, version 9.3 (SAS Institute), and STATA, version 14.2 (StataCorp).

## Results

We were able to reach 369 eligible patients by telephone, of whom 105 declined to participate. We therefore administered a telephone survey to 264 participants, of whom 53% were white, 29% were Asian American, and 5% African American women. Thirty-six percent of women were of Latino/Hispanic ethnicity. The mean age of women in the analytic sample was ∼37 years (Table 1). In terms of education, 31% had a postgraduate degree (*n* = 81), 41% were college graduates (*n* = 107), and 29% did not graduate from college (*n* = 76). The mean A1c for our sample was 5.7% (standard deviation = 0.3), with no significant differences between groups of different educational attainment (*p* = 0.24). Participants were obese, on average, with a mean BMI of 30.3 kg/m^2^, with the highest values among women who did not graduate from college (31.8 mg/m^2^, vs. 29.6 kg/m^2^ for college graduates and 29.7 kg/m^2^ for women with a postcollege degree, *p* = 0.04). Survey participants and survey nonrespondents were similar in mean age (37.4 vs. 38.2 years), with identical mean A1c and BMI in both groups.

**Table 1.** Demographic and Clinical Characteristics of Study Sample, by Educational Attainment (*n* = 264)

	Less than college graduate (*N* = 76)	College graduate (*N* = 107)	Postcollege degree (*N* = 81)	*p*
Mean age (SD)	36.2 (5.3)	38.1 (4.4)	37.6 (4.0)	**0.021**
Race, %				
White	67.1	43.0	53.1	**<0.01**
African American	6.6	3.7	6.2	
Asian/Pacific Islander	6.6	42.1	33.3	
Other	14.5	8.4	6.2	
Missing	5.3	2.8	1.2	
Ethnicity, %				
Latino/Hispanic	68.4	26.2	19.8	**<0.01**
Not Latino/Hispanic	31.6	72.0	80.2	
Missing	—	1.9	—	
Number of children (SD)	2.2 (1.3)	1.9 (0.9)	1.8 (1.3)	0.07
% w/family history of DM, %	80.0	64.4	64.1	**0.05**
Mean BMI (kg/m^2^)	31.8 (6.4)	29.6 (4.8)	29.7 (5.2)	**0.04**
HbA1c (%)	5.73 (0.29)	5.71 (0.35)	5.62 (0.34)	0.24
“I don't think I truly had gestational diabetes,” %				
Agree	35.5	29.9	32.1	0.72
Disagree	64.5	70.1	67.9	
Interest in taking MTF to prevent T2DM, %				
Not interested	58.1	63.8	58.8	0.68
Interested	41.9	36.2	41.2	
Interested in attending DPP classes, %				
Not interested	29.3	43.9	43.8	0.10
Interested	70.7	56.1	56.2	
Any interest in DPP and/or MTF, %				
DPP yes/MTF yes	41.1	24.8	25.3	**0.03**
DPP yes/MTF no	28.8	32.4	30.4	
DPP no/MTF yes	1.4	11.4	16.5	
DPP no/MTF no	28.8	31.4	27.8	
Amount willing to pay for DPP (SD)	$125 (211)	$195 (258)	$167 (176)	0.24
Decision-making preferences, %				
I make the decision	18.4	28.0	38.3	**<0.01**
I share responsibility	63.2	67.3	51.8	
Leave decisions to MD	18.4	4.7	9.9	

Bold values indicate statistically significant results (*p* < 0.05).

BMI, body mass index; DM, diabetes mellitus; DPP, Diabetes Prevention Program; HbA1c, hemoglobin A1c; MD, medical doctor; MTF, metformin; SD, standard deviation; T2DM, type 2 diabetes mellitus.

Women who did not graduate from college also had the highest reported rates of a family history of type 2 diabetes (80%, vs. 64% each for college graduates and women with a postcollege degree, *p* = 0.05). There were no differences between the three education groups in agreement with the statement “I don't think I truly had gestational diabetes” (*p* = 0.72), or, among the subgroup of women who expressed interest in participating in DPP classes, in the out-of-pocket cost women were willing to pay (*p* = 0.24). Women across the entire sample were theoretically willing to pay an average of $130 for the 12-month program, much less than the true cost of providing the DPP of ∼$500 per participant without insurance coverage. Women who specifically expressed interest in the DPP were theoretically willing to pay an average of $164 for the 12-month program (data not shown).

In unadjusted analyses more than 80% of women preferred to make medical decisions themselves or together with their provider, regardless of educational attainment (Table 1). However, in multivariate analyses adjusted for age, race, and ethnicity, women who did not graduate from college were more likely to leave medical decisions entirely to their provider (*p* < 0.01) compared to women with a postgraduate degree (Table 2). We did not find a significant difference in medical decision-making preferences when comparing women who graduated from college and those with a postcollege degree (*p* = 0.06). We also found no difference in interest in DPP lifestyle change when comparing women who did not graduate from college (odds ratio [OR] 1.67, *p* = 0.09) or college graduates (OR 0.89, *p* = 0.17) with women who had a postcollege degree (Table 3). Similarly, we found no difference in interest in metformin when comparing women who did not graduate from college (OR 1.04, *p* = 0.49) or college graduates (OR 0.68, *p* = 0.16) with women who had a postcollege degree (Table 4).

**Table 2.** Multivariate Analysis of Educational Attainment and Preference to be Involved in Final Medical Decisions among Women with a History of Gestational Diabetes Mellitus

Variable	OR (95% CI)	*p*
Postcollege degree (reference)		
College graduate	0.91 (0.50–1.6)	0.06
Less than college graduate	0.29 (0.14–0.61)	**<0.01**

Adjusted for age, race, ethnicity. Significant *p*-value (<0.05) in bold font.

CI, confidence interval; OR, odds ratio.

**Table 3.** Multivariate Analysis of Educational Attainment and Interest in Diabetes Prevention Program Lifestyle Change among Women with a History of Gestational Diabetes Mellitus

Variable	OR (95% CI)	*p*
Postcollege degree (reference)		
College graduate	0.89 (0.49–1.62)	0.17
Less than college graduate	1.67 (0.80–3.46)	0.09

Adjusted for age, race, ethnicity.

**Table 4.** Multivariate Analysis of Educational Attainment and Interest in Metformin among Women with a History of Gestational Diabetes Mellitus

Variable	OR (95% CI)	*p*
Postcollege degree (reference)		
College graduate	0.68 (0.37–1.28)	0.16
Less than college graduate	1.04 (0.50–2.13)	0.49

Adjusted for age, race, ethnicity.

## Discussion

Within an insured patient population, we found that most women with a GDM history preferred to make decisions with their physician, regardless of educational level. Specifically, while there were statistical differences between groups, more than 80% of women who did not graduate from college and more than 90% of women who had a postcollege degree wanted to actively participate in medical decisions. Interest in metformin or nonpharmacologic (*i.e.*, DPP lifestyle change) approaches to type 2 diabetes prevention did not vary by educational attainment, contrary to our original hypotheses. Our findings underscore the importance of presenting both evidence-based type 2 diabetes prevention options to women who are high risk due to a history of GDM irrespective of their educational attainment.

There are numerous challenges to successful engagement of women with a GDM history in evidence-based type 2 diabetes prevention in the real world. Denial and limited understanding of personal risk are important first-level barriers to wider uptake of type 2 diabetes prevention in this population. In one study, almost 25% of women diagnosed with GDM did not believe this diagnosis.^21^ Our analysis showed similar findings, with 30%–35% of women not agreeing that they truly had GDM. Disagreement with the GDM diagnosis was similar for women with a postcollege degree and women who did not graduate from college, indicating that formal education does not necessarily translate to understanding and acceptance of oral glucose tolerance testing and other objective screening measures for GDM. Other work has found that while 90% of women with a GDM history were aware that this diagnosis was a risk factor for type 2 diabetes mellitus (T2DM), only 20% believed that they were personally at high risk.^22^ There is a need to communicate to all women with a GDM history regardless of educational attainment that they themselves are at risk for T2DM, that the risk data are not impersonal statistics, and that there are proven strategies to help them reduce their diabetes risk, with potential health benefits for themselves and their families.^23^

Even women with a GDM history who accept the diagnosis and are fully aware of their risk for progression to type 2 diabetes are unlikely to engage in evidence-based diabetes prevention. Women with young children often report stress, fatigue, and time and financial constraints associated with parenthood, which can make uptake of and adherence to lifestyle interventions extremely challenging.^9–12^ Metformin is a similarly effective, evidence-based option for overweight/obese women with a history of GDM, but to our knowledge, this is the first published study examining perceptions of metformin use for diabetes prevention among women with a GDM history. In our analysis, ∼55%–70% of women expressed interest in DPP lifestyle change regardless of educational attainment, while ∼35%–40% expressed interest in metformin. The high levels of interest in uptake of evidence-based prevention strategies, as well as a strong preference among these women to participate in medical decisions, suggest that SDM may be an ideal approach to promote uptake and engagement in type 2 diabetes prevention strategies among women with a GDM history.

There are few studies specifically examining the relationship between educational attainment and its influence on the feasibility and success of SDM. Concerns have been raised that SDM will be more acceptable to and more effective in patients who are highly educated with high health literacy. For instance, patients from low- and middle-income countries are less likely to engage in SDM for reasons that may include limited awareness of their medical condition and differing cultural beliefs.^24^ However, a recent meta-analysis found that SDM is more beneficial for patients with low health literacy compared to patients with high health literacy.^25^ Future intervention studies engaging women with a GDM history in SDM for type 2 diabetes prevention should include women across the spectrum of educational attainment and should not exclude women with low health literacy.

Our team recently published work showing that a pharmacist-led SDM intervention was associated with a higher uptake of either DPP lifestyle change or metformin and greater weight loss, but only 4 of the 287 female study participants had a history of GDM.^14^ To our knowledge, no diabetes prevention interventions among women with a history of GDM have included SDM.^16–18^ Studies of SDM in type 2 diabetes focused on self-management decisions that have demonstrated the benefits of this approach, including better diabetes self-management, increased knowledge of their disease process, and increased patient confidence.^26,27^ There are several reasons why an approach using SDM in these women with a GDM history may be beneficial, including the opportunity to educate patients on the growing number of virtual DPP lifestyle change programs that are available. Virtual lifestyle change programs have shown benefits in terms of weight loss for a broader population with prediabetes and may be particularly attractive to women with young children and/or time constraints.^28^

We found that even women with a postcollege degree who presumably have higher household incomes were only theoretically willing to pay approximately one-third of the “true” cost of providing the DPP curriculum. Efforts to engage women with a GDM history in diabetes prevention, including SDM, will only be successful if insurance plans, workplaces, or other stakeholders with an interest in diabetes prevention continue to cover most of the up-front cost of the DPP for this high-risk population.

This study has several limitations. All survey participants had health insurance and only 5% were insured by Medicaid, so our findings are unlikely to generalize to uninsured and/or low-income populations. We identified women as having had a history of GDM based on billing codes and were not able to review oral glucose tolerance tests to confirm this diagnosis for each patient. We were unable to confirm specific DPP insurance coverage details for each survey participant, so our question about the amount participants were willing to pay for the DPP was theoretical. Finally, social desirability bias may have influenced women to overestimate their interest in evidence-based diabetes prevention. However, we combined responses of “somewhat,” “moderately,” and “very” interested in either DPP lifestyle change or metformin into single composite outcome variables to try and attenuate response bias.

In summary, our study found that within an insured population, differences in educational attainment among women with a GDM history are not associated with differences in perceived risk of progression to type 2 diabetes or associated with interest in either of the two evidence-based options for diabetes prevention, namely DPP lifestyle change or metformin. Most women with a GDM history prefer to be involved in the decision about which diabetes prevention option/s would be best for them, and the DPP confirmed that these two options have similar efficacy among this population. There is a need for future intervention studies that engage these women across levels of educational attainment in personalized discussions about both options, ideally using an SDM approach, which measure outcomes, including uptake of an evidence-based diabetes prevention strategy, percentage of weight loss, and ultimately a decrease in the incidence of type 2 diabetes.

## Abbreviations Used

BMI: body mass index
CDC: Centers for Disease Control and Prevention
CI: confidence interval
DM: diabetes mellitus
DPP: Diabetes Prevention Program
EHR: electronic health record
GDM: gestational diabetes mellitus
MD: medical doctor
MTF: metformin
OR: odds ratio
SD: standard deviation
SDM: shared decision-making
T2DM: type 2 diabetes mellitus
UCLA: University of California, Los Angeles
